# Assessment of the Relationship Between Internet Addiction, Psychological Well-Being, and Sleep Quality: A Cross-Sectional Study Involving Adult Population

**DOI:** 10.3390/bs15030344

**Published:** 2025-03-11

**Authors:** Mehmet Emin Arayici, Sema Gultekin Arayici, Ozum Erkin Geyiktepe, Hatice Simsek

**Affiliations:** 1 Department of Public Health, Faculty of Medicine, Dokuz Eylül University, Inciralti-Balcova, Izmir 35340, Turkey; 2 Department of Biostatistics and Medical Informatics, Faculty of Medicine, Dokuz Eylül University, Inciralti-Balcova, Izmir 35340, Turkey; 3 Department of Clinical Psychology, Institute of Social Sciences, Ege University, Bornova, Izmir 35040, Turkey; 4 Department of Public Health Nursing, Faculty of Health Sciences, Izmir Democracy University, Karabağlar, Izmir 35140, Turkey

**Keywords:** internet addiction, sleep quality, psychological well-being, adults, socio-demographic factors

## Abstract

Internet addiction is an emerging public health concern among adults, potentially affecting psychological well-being and sleep quality. Although a substantial body of research has focused on adolescents and younger adults, less is known about middle-aged and older adult populations. This study investigated the relationships between Internet addiction, sleep quality, and psychological well-being in 629 adults (aged 30–60 years) and examined the socio-demographic predictors of Internet addiction. Participants completed online questionnaires assessing Internet addiction, psychological well-being, and sleep quality (Pittsburgh Sleep Quality Index). The final sample had a mean age of 39.4 (SD = 7.8), with 53.4% female participants. Most were employed (77.9%), and nearly half held an undergraduate degree (49.1%). The mean Internet addiction score was 38.1 ± 13.6. Poor sleep quality was prevalent (67.2%), and Internet addiction was positively correlated with total PSQI scores (r = 0.593; *p* < 0.001). Higher psychological well-being was inversely associated with both Internet addiction (r = −0.417; *p* < 0.001) and poor sleep quality (r = −0.490; *p* < 0.001). Younger age, female gender, regular employment, and higher income predicted greater Internet addiction, whereas having an undergraduate degree was associated with lower scores. Taken together, the findings of this study emphasize the importance of addressing sleep quality and psychological well-being to mitigate excessive Internet use in mid-life and older populations, particularly among those at higher risk.

## 1. Introduction

It is a well-known fact that the rapid expansion of digital technology has significantly transformed modern lifestyles, with the Internet becoming an indispensable component of everyday life (Hammad et al., 2024; Wang et al., 2024). This pervasive integration of technology, while catalyzing advancements in information accessibility and connectivity, has concurrently given rise to phenomena such as Internet addiction (Wang et al., 2024). Internet addiction is characterized by compulsive online behaviors that interfere with daily functioning and may lead to detrimental effects on mental and physical health (Jiang, 2019). The escalating incidence of Internet addiction among adults has elicited scholarly concern, particularly regarding its potential to compromise psychological well-being and deteriorate sleep quality (Ozuysal et al., 2024). As the digital landscape continues to evolve, understanding the multifaceted implications of excessive Internet use is imperative for both theoretical advancement and the formulation of effective public health strategies (Ayran et al., 2019).

In recent years, psychological well-being has garnered increasing attention within the realm of mental health research (Salunkhe et al., 2022; Yang, 2024). Psychological well-being is a multifaceted construct that encompasses emotional balance, life satisfaction, and effective coping mechanisms in the face of daily stressors (Chiorean, 2023). Previous studies have linked excessive Internet use to various negative psychological outcomes, including heightened levels of anxiety, depression, and social isolation (Salunkhe et al., 2022). This growing body of literature suggests that the compulsive use of digital platforms might undermine aspects of psychological well-being, necessitating a deeper exploration into these interrelations (Mihajlović et al., 2008). Sleep quality represents another critical dimension of health that may be adversely affected by excessive Internet use (Kumar Swain & Pati, 2021). Adequate sleep is essential for cognitive functioning, emotional regulation, and overall physical health (Singh et al., 2021). However, the pervasive use of Internet-enabled devices, especially during evening hours, has been associated with disruptions in circadian rhythms and a reduction in sleep quality (Barath et al., 2020; Kang et al., 2015). The interplay between prolonged screen time, blue light exposure, and mental preoccupation with online content may contribute to sleep disturbances, thereby creating a cycle of impaired mental health and diminished well-being (An et al., 2014).

Despite the increasing scholarly attention to Internet addiction, there remains a significant gap in the literature regarding its comprehensive effects on psychological well-being and sleep quality among adults (Kinsella & Chin, 2024; Raj, 2021). Most existing research has focused on adolescent populations or has examined these factors in isolation (Bourhis et al., 2012; Paudel et al., 2021; Soriano-Molina et al., 2025). For example, recent studies have consistently shown a significant negative relationship between Internet addiction and psychological well-being among young adults and college students (Bali & Bakhshi, 2020; Çardak, 2013; Fantaw, 2020; Sharma & Sharma, 2018). Internet addiction has also been associated with poor sleep quality (Andhi et al., 2022; Begum, 2024; Raj, 2021), with one study finding that 87% of participants with Internet addiction exhibited poor sleep quality (Andhi et al., 2022). The prevalence of Internet addiction among students ranged from 28.2% to 45.1% across studies (Begum, 2024; Fantaw, 2020). Excessive Internet use, particularly for entertainment purposes and for more than six hours daily, was linked to lower psychological well-being and increased risk of Internet addiction (Fantaw, 2020). By integrating the investigation of psychological well-being and sleep quality with Internet addiction, the present study aims to provide a more holistic understanding of how excessive digital engagement can affect overall adult health (Gupta & Kaura, 2019). This integrated approach is essential for developing targeted interventions that address both behavioral and psychological components of Internet use (Ozuysal et al., 2024; Sudharkodhy et al., 2022). However, despite these consistent findings, the existing literature presents certain inconsistencies and gaps that warrant further investigation. For instance, while several studies have reported a strong negative association between Internet addiction and psychological well-being (Bali & Bakhshi, 2020; Çardak, 2013; Kaur & Kaur, 2017; Sharma & Sharma, 2018), others have suggested that this relationship may be moderated by factors such as personality traits, coping strategies, or social support (Chwaszcz et al., 2018; Lu et al., 2023). Similarly, the impact of Internet addiction on sleep quality is not uniform across all populations, as some studies indicate that the severity of sleep disturbances varies depending on age, screen exposure duration, and the type of online activity (Agarwal et al., 2020; ElSalhy et al., 2019). Moreover, an important limitation of prior research is its predominant focus on adolescent and student populations, raising questions about the generalizability of these findings to broader adult populations. Given that adults may experience different patterns of Internet use, stressors, and lifestyle factors, it remains unclear whether the same associations hold in older age groups or among individuals with varying occupational and socioeconomic backgrounds. Addressing these gaps will provide a clearer understanding of how Internet addiction affects psychological and physiological health outcomes, ultimately informing more targeted and effective intervention strategies.

Recent scholarship on the intersection of Internet addiction, sleep quality, and psychological well-being has highlighted the need for a more cohesive theoretical framework to anchor empirical findings. In this regard, the Problematic Internet Use Model (Caplan, 2010) offers critical insights by proposing that an individual’s preference for online social interaction can foster deficient self-regulation, ultimately triggering negative outcomes such as compromised sleep patterns and reduced well-being. Complementing this perspective is the Compensatory Internet Use Theory (Kardefelt-Winther, 2014), which posits that some individuals may rely on excessive Internet use to cope with stress or unmet needs in their offline lives. While such compensatory behavior may temporarily alleviate distress, it can also perpetuate maladaptive outcomes when digital engagement becomes a substitute for healthier coping strategies.

In light of the pervasive influence of the Internet on contemporary life, it is imperative to explore the potential adverse consequences of its overuse on mental health and sleep patterns. This study is designed to evaluate the relationship between Internet addiction, psychological well-being, and sleep quality among adults, thereby contributing to a more nuanced understanding of the digital age’s impact on health. The findings are expected to inform both clinical practices and public health policies, promoting strategies that mitigate the negative outcomes associated with excessive Internet usage.

## 2. Methods

### 2.1. Study Design and Setting

This study was designed as a community-based cross-sectional investigation. Although the study was conceptualized in İzmir, data were collected through data forms administered across various regions of Turkey to capture a diverse sample of the adult population. Data were gathered between November 2024 and January 2025, ensuring representation from various regions and demographic groups across Türkiye. The study was conducted in accordance with the Declaration of Helsinki and was approved by the Institutional Review Board of the Dokuz Eylul University Faculty of Medicine Non-Interventional Research Ethics Committee, with decision number 2024/37-11, on 6 November 2024. All of the participants provided informed consent prior to participating in the study.

The target population consisted of adults aged 30 years and older residing in Türkiye. The sample size was determined based on the anticipated proportion of the general population possessing Internet addiction. It was calculated using the single-sample proportion formula, n = [(Z α/2)2 P (1 − P)/d2], where n is the required sample size, Zα/2 corresponds to the Z-value for a 95% confidence interval, d represents a 5% margin of error, and a statistical power of 80% (1 − β = 0.80) was assumed. Since the frequency of Internet addiction in the adult population was unknown, a value of 50% was used for *p* as an estimated response distribution. Under these assumptions, the minimum sample size required to ensure statistical robustness was calculated to be at least 385 participants.

### 2.2. Inclusion and Exclusion Criteria

The inclusion criteria for the study required that participants be aged 30–60 years, voluntarily agree to participate, and provide informed consent. The study targeted individuals residing within the borders of Turkey, and it was also necessary that participants possessed sufficient proficiency in the survey language to comprehend and respond to the questionnaire adequately. Furthermore, participants were required to be capable of completing the survey, meaning that no cognitive limitations that could impede their ability to respond were present. Individuals who did not provide consent or who completed less than half of the survey items were excluded from the study.

### 2.3. Data Collection Procedures and Instruments

Potential participants were approached through community centers, university campuses, and online social media platforms. Individuals who volunteered to participate were provided with an overview of the study’s objectives and procedures. Before completing the questionnaire, each participant was required to review and sign an informed consent form outlining the purpose of the study, the voluntary nature of participation, the expected time commitment, and assurances of confidentiality and anonymity. Data were collected either in person or through a secure online platform. In the in-person setting, participants completed a paper-based version of the questionnaire in a quiet space. In the online setting, participants accessed the digital questionnaire via a secure link and were instructed to complete it in one sitting without interruptions. Clear written instructions were provided to ensure accurate and independent responses. To protect participants’ identities, all of the questionnaires were anonymized by assigning unique identifier codes. The data were stored in a password-protected electronic database accessible only to the principal investigator and authorized research team members. Upon completion, each questionnaire was reviewed for missing responses or obvious patterns of non-engagement (e.g., the same response across all items). In cases of incomplete submissions, participants were contacted, when feasible, to clarify missing data. Incomplete questionnaires that could not be verified were excluded from the final analysis. The data were gathered using a structured questionnaire composed of three standardized scales and a demographic information form.

#### 2.3.1. Demographic Information Form

A structured demographic form was used to assemble information on participants’ background characteristics. Specifically, they were asked to indicate their year of birth, gender as female or male, and marital status as married, single, or in a partnership. They also reported their highest educational attainment as non-literate, primary school graduate, middle school graduate, high school graduate, undergraduate, and postgraduate. Smoking was categorized as current or no smoking, and alcohol use was categorized as drinking alcohol once or more than once a month. Additionally, participants provided details about their occupation and employment status, specifying whether they were currently working in a job that provides a regular income or not employed at the time. Finally, they were asked to evaluate their income level in relation to their expenditures, stating whether their income was greater than, equal to, or less than their expenses.

#### 2.3.2. The Internet Addiction Scale

The Internet Addiction Scale was developed by Hahn and Jerusalem (2001) and subsequently adapted into Turkish by Şahin and Korkmaz (2011). The scale comprises 19 items, and the Cronbach’s alpha reliability coefficient for the scale was reported as 0.85. It is a 5-point Likert-type scale, where 1 corresponds to “never” and 5 corresponds to “always”. Higher scores indicate a higher level of Internet addiction. The scale is intended for use with adult populations.

#### 2.3.3. Pittsburgh Sleep Quality Index

To assess participants’ sleep quality, this study employed the Pittsburgh Sleep Quality Index (PSQI), originally developed by Buysse et al. (1989) (Cronbach’s α = 0.80) and later adapted into Turkish by Ağargün et al. in 1996 (Ağargün et al., 1996) (Cronbach’s α = 0.80). The instrument consists of the following seven subcomponents: subjective sleep quality, sleep latency, sleep duration, habitual sleep efficiency, sleep disturbances, use of sleeping medication, and daytime dysfunction. Total scores range from 0 to 21, with a PSQI score below 4 indicating good sleep quality and a score of 5 or higher signifying poor sleep quality.

#### 2.3.4. Psychological Well-Being Scale

Developed by Diener et al. in 2010 (Diener et al., 2010) to complement existing measures of well-being and to capture socio-psychological well-being, the Psychological Well-Being Scale was adapted into Turkish by Telef (Telef, 2013). Exploratory factor analysis revealed that the scale accounted for 42% of the total variance, with factor loadings for individual items ranging between 0.54 and 0.76. The internal consistency of the scale, as measured by Cronbach’s alpha, was reported to be 0.80. Furthermore, the test–retest analysis indicated a high-level, positive, and significant correlation between the first and second administrations (r = 0.86; *p* < 0.001). The scale items are rated on a 1–7 Likert-type format, ranging from “strongly disagree” (1) to “strongly agree” (7), with all items expressed in a positive manner. Total scores range from 8 (if a respondent selects “strongly disagree” for all items) to 56 (if a respondent selects “strongly agree” for all items). A higher score indicates that the individual possesses greater psychological resources and strengths.

### 2.4. Statistical Analysis

Descriptive statistics were used to summarize the socio-demographic characteristics of the participants, including means and standard deviations (mean ± SD) for continuous variables, and frequencies and percentages for categorical variables. The normality of continuous data was assessed using the Kolmogorov–Smirnov test, and Skewness and Kurtosis symmetric distributions of the data. To examine the predictors of Internet addiction scores, a multiple linear regression analysis was conducted. The backward stepwise elimination method was applied to refine the model by retaining only significant predictors. Dummy variables were created for categorical variables, including gender (reference: female), working status (reference: unemployed), and income status (reference: “my income is less than my expenses”). The final model included significant predictors such as age, gender, working status, educational status, income status, and Pittsburgh Sleep Quality Index (PSQI) scores. Regression coefficients (β), standard errors, 95% confidence intervals (CIs), and *p*-values were reported for each predictor. The goodness-of-fit of the model was evaluated using the coefficient of determination (R^2^), and the statistical significance of the model was assessed using the F-test. All statistical analyses were conducted using STATA software (v.18, College Station, TX, USA) and the SPSS (v30.0) package program, and statistical significance in all of the analyses was determined at a two-tailed *p*-value of <0.05.

## 3. Results

**Figure 1:**
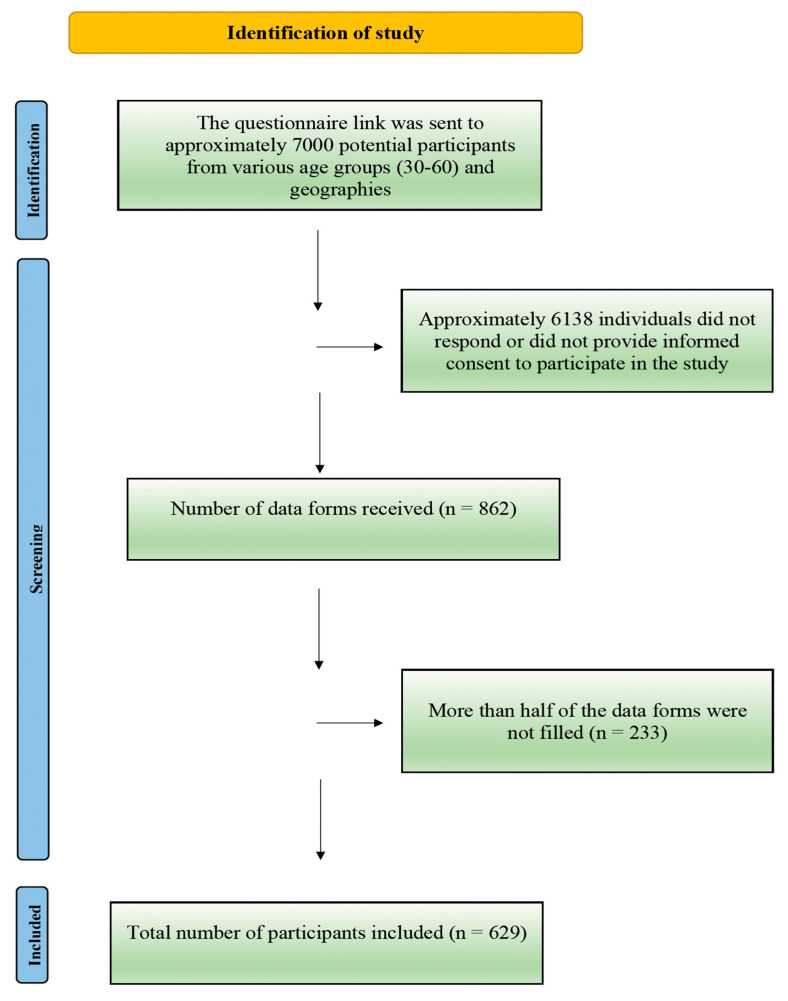
Flow diagram showing the assignment process of the participants for the study.

A questionnaire link was distributed to approximately 7000 potential participants from diverse age groups (aged 30–60 years) and geographical locations in Türkiye. Out of these, 6138 individuals either did not respond or declined to provide informed consent to participate. Consequently, 862 data forms were initially received. However, 233 of these forms were excluded due to being incomplete or insufficiently filled. Ultimately, the study included 629 participants who completed the data forms adequately. A total of 629 participants were included in the study, and a flowchart illustrating the participant’s selection process is summarized in Figure 1.

The baseline descriptive and socio-demographic characteristics of the study group, which consisted of 629 participants, is presented in Table 1. The mean age of the participants was 39.4 years (SD = 7.8; min–max = 30–60). Regarding the gender distribution, 53.4% of the participants were female, while 46.6% were male. In terms of relationship status, 29.9% of the participants were single and 70.1% were in a relationship. Educational status varied, with 33.1% having a high school education or lower, 49.1% holding an undergraduate degree, and 17.8% possessing a postgraduate degree. For working status, 22.1% of the participants reported not working, while 77.9% stated they were employed in a job that provides regular income. Income status showed that 40.1% of participants indicated their income was less than their expenses, 33.5% reported their income was equal to their expenses, and 26.4% said their income exceeded their expenses. Additionally, 24.2% of participants were smokers and 75.8% were non-smokers. Regarding alcohol consumption, 40.3% reported consuming alcohol at least once a month, while 59.7% did not consume alcohol.

A wide variety of professions are represented, reflecting the diverse socio-demographic characteristics of the sample. Teachers constituted the largest group, accounting for 14.9% of the participants (n = 94). This was followed by nurses (10.7%, n = 67) and physiotherapists (4.1%, n = 26). Other notable occupational groups included municipal workers (4.0%, n = 25), retirees (3.8%, n = 24), and manual laborers (3.7%, n = 23). The sample also included diverse proportions of professionals such as academics (2.5%, n = 16), psychologists (2.4%, n = 15), and various specialists in healthcare, education, and sports-related fields (not presented as a table).

The mean score for Internet addiction was 38.1 ± 13.6, with scores ranging from 19 to 95 (Table 2). Psychological well-being was measured with an 8-item scale, yielding an average score of 43.2 ± 8.8 (range: 8–56). Sleep quality was assessed using the PSQI, with a mean total score of 6.9 ± 4.0 (range: 0–19) (Table 2). Subscale scores for the PSQI were as follows: subjective sleep quality (1.3 ± 0.8), sleep onset latency (1.2 ± 0.9), sleep duration (0.7 ± 1.0), habitual sleep efficiency (0.9 ± 1.1), sleep disorders (1.4 ± 0.6), use of sleep medication (0.2 ± 0.8), and daytime dysfunction (1.0 ± 0.9), each with a possible range of zero to three. The analysis of the overall sleep quality showed that 32.8% of participants were classified as having good sleep quality (PSQI < 4), while 67.2% were categorized as having poor sleep quality (PSQI ≥ 5) (Table 2).

Table 3 presents the correlation coefficients (r) and *p*-values for the relationships between Internet addiction, psychological well-being, and the Pittsburgh Sleep Quality Index (PSQI) total and subscale scores among the study participants (n = 629). Internet addiction was found to be positively correlated with the PSQI total score (r = 0.593; *p* < 0.001) and all of its subscales, indicating that higher levels of Internet addiction were associated with poorer sleep quality, as reflected by increased scores in subjective sleep quality (r = 0.411; *p* < 0.001), sleep onset latency (r = 0.434; *p* < 0.001), sleep duration (r = 0.377; *p* < 0.001), habitual sleep efficiency (r = 0.141; *p* < 0.001), sleep disorders (r = 0.411; *p* < 0.001), use of sleep medication (r = 0.429; *p* < 0.001), and daytime dysfunction (r = 0.522; *p* < 0.001). Conversely, psychological well-being showed a significant negative correlation with Internet addiction (r = −0.417; *p* < 0.001) and the PSQI total score (r = −0.490; *p* < 0.001), as well as all of the PSQI subscales except habitual sleep efficiency (*p* = 0.203). This suggests that higher psychological well-being is associated with lower Internet addiction and better sleep quality.

As illustrated in Table 4, the multiple linear regression analysis evaluated the factors influencing Internet addiction scores, revealing that 52% of the variance in Internet addiction was explained by the model (R = 0.721; R^2^ = 0.520). Age was found to be negatively associated with Internet addiction (β = −0.184; *p* < 0.001), indicating that older participants had lower levels of Internet addiction. Gender also had a significant impact, with males reporting lower scores compared to females (β = −0.088; *p* = 0.004). Participants who were employed in regular jobs showed higher Internet addiction scores (β = 0.082; *p* = 0.019), while those with an undergraduate degree had significantly lower scores compared to other educational groups (β = −0.072; *p* = 0.024). Income status was a notable predictor, as participants whose income was equal to (β = 0.176; *p* < 0.001) or greater than (β = 0.136; *p* < 0.001) their expenses exhibited higher levels of Internet addiction. Sleep quality, as assessed by the PSQI, was a significant contributor to Internet addiction scores. Poor overall sleep quality (PSQI total score, β = 1.067 and *p* < 0.001) was associated with increased Internet addiction. Among the PSQI subscales, subjective sleep quality (β = −0.216; *p* < 0.001), habitual sleep efficiency (β = −0.352; *p* < 0.001), and sleep duration (β = −0.133; *p* = 0.007) were negatively associated with Internet addiction, suggesting that better sleep in these dimensions reduced the addiction scores.

## 4. Discussion

The present study sought to investigate the interplay between Internet addiction, sleep quality, and psychological well-being in a diverse sample of 629 adults from various regions in Türkiye. By targeting individuals aged 30 and above, this research aimed to capture a segment of the population that may often be overlooked in studies focusing primarily on adolescents or emerging adults. Additionally, the demographic characteristics—encompassing gender balance, varied income levels, and diverse educational backgrounds—allowed for a nuanced examination of how socio-demographic variables influence and potentially exacerbate Internet-related behaviors. Previous literature has extensively documented the rising prevalence of Internet addiction across different age groups; however, evidence remains mixed regarding the strength and direction of its relationship with both sleep quality and mental health in mid-life populations. Given the shifts in occupational structures, expanding digital landscapes, and changing social norms, it is crucial to identify risk factors as well as protective elements in this older cohort. The current findings, indicating a moderate mean Internet addiction score (38.1 ± 13.6) and substantial differences in sleep quality (with 67.2% classified as poor sleepers), underscore the need to address how digital engagement patterns and psychological well-being intersect. By focusing on correlations among key variables and employing multiple linear regression to detect potential predictors, this study advances our understanding of why certain subgroups may be more vulnerable to the adverse outcomes linked to excessive Internet use.

A key finding was the robust positive correlation between Internet addiction and poorer sleep quality (r = 0.593; *p* < 0.001) (Bhagirath Sonawane et al., 2024). This is consistent with prior studies indicating that excessive Internet use—particularly at night—tends to disrupt circadian rhythms, reduce total sleep time, and impair the overall quality of rest (Wang et al., 2024). Given the high proportion of individuals identified with poor sleep quality (67.2%), these results emphasize the potential negative impact of problematic Internet use on both sleep duration and sleep efficiency (Aldea-Parra et al., 2024). Moreover, poor sleep quality linked to excessive Internet use has been associated with daytime dysfunction, decreased cognitive performance, and heightened levels of stress and anxiety (Ozuysal et al., 2024). The findings highlight the urgency of addressing problematic Internet use as a public health concern. Implementing interventions such as digital detox programs, awareness campaigns, and policies limiting screen exposure before bedtime may help mitigate the adverse effects on sleep (Fareeq Saber et al., 2024).

The negative association between psychological well-being and Internet addiction (r = −0.417; *p* < 0.001) underscores the possible detrimental effects of compulsive online behaviors on mental health (Kumar et al., 2024). This finding aligns with previous studies indicating that excessive Internet use is linked to heightened levels of anxiety, depression, and stress, which collectively contribute to lower psychological well-being (Boukadida et al., 2023). Moreover, Internet addiction has been found to disrupt social relationships, reduce real-world interactions, and foster feelings of loneliness and emotional instability (Nikitchenko & Koroteeva, 2023). These detrimental effects may be particularly pronounced among young adults and university students, who often rely on digital platforms for academic and social purposes but are also at a higher risk of compulsive Internet use (Zahid & Rauf, 2022). Furthermore, the inverse relationship between psychological well-being and the PSQI total score (r = −0.490; *p* < 0.001) suggests that suboptimal mental health may exacerbate sleep disturbances, creating a cycle in which poor psychological well-being and insufficient sleep reinforce one another (Jehangir, 2022). Individuals with high levels of Internet addiction often experience irregular sleep patterns, shorter sleep duration, and increased nocturnal awakenings, all of which negatively impact their emotional regulation and cognitive function (Hussien, 2022). Additionally, poor sleep quality has been linked to higher stress sensitivity and emotional instability, further intensifying the psychological burden associated with excessive Internet use (M. He et al., 2024). This interplay between Internet addiction, mental distress, and sleep disturbances highlights the importance of targeted interventions, such as digital detox strategies, mindfulness training, and cognitive behavioral therapy, to break this negative cycle and improve overall well-being (Rajak & James, 2023).

The multiple linear regression analysis offered deeper insights into the predictors of Internet addiction. Notably, age showed a negative association with Internet addiction (β = −0.184; *p* < 0.001), suggesting that younger individuals might be at higher risk for problematic Internet use (Pjevac et al., 2024). This is consistent with findings indicating that adolescents and young adults, particularly those under 25 years old, exhibit higher rates of Internet addiction due to their increased engagement in online activities such as social media, gaming, and streaming (Dutta & Karmakar, 2024). Furthermore, research suggests that, as individuals age, their Internet consumption patterns shift toward more purpose-driven and regulated use, reducing the likelihood of compulsive Internet behaviors (Pjevac et al., 2024). The significant effect of gender, with males reporting lower scores compared to females (β = −0.088; *p* = 0.004), highlights potential gender differences in the patterns or types of online activities that contribute to addictive behaviors (Varchetta et al., 2024). While men are more likely to engage in Internet gaming disorder and compulsive online gambling, women exhibit a greater tendency toward social media and smartphone overuse, contributing to differences in Internet addiction prevalence and severity (Ponizovsky et al., 2022). Additionally, studies indicate that gender-related psychological factors, such as emotional regulation and reward dependence, may play a role in shaping online behaviors and the risk of developing problematic Internet use (Swain et al., 2024). These demographic factors warrant further exploration to develop targeted prevention strategies, including gender-specific interventions that address distinct behavioral patterns and online engagement tendencies (Kalawat et al., 2024).

Educational and employment statuses also emerged as important determinants. Those holding an undergraduate degree exhibited significantly lower Internet addiction scores (β = −0.072; *p* = 0.024), possibly reflecting a greater awareness of balanced Internet use or more access to alternative social and intellectual pursuits (Bakalis et al., 2023). Studies suggest that higher levels of education are associated with more responsible Internet usage, as individuals with university degrees are more likely to integrate the Internet into their professional and academic activities rather than for compulsive recreational use (Mihăilescu, 2023). Moreover, a structured educational environment can provide individuals with healthier habits and a greater sense of time management, reducing their risk of developing problematic Internet behaviors (Zhukova et al., 2023). By contrast, being employed in a regular job was associated with higher Internet addiction scores (β = 0.082; *p* = 0.019), suggesting that structured work routines do not necessarily guard against problematic Internet use (S. He & Chen, 2024). This finding aligns with research indicating that workplace stress and digital work environments can contribute to excessive screen time and compulsive online behaviors (Zengin & Naktiyok, 2022). In certain professions, especially those requiring prolonged Internet use, employees may struggle with boundaries between work-related online activities and recreational digital engagement, increasing the risk of Internet addiction (Setko et al., 2024). It is conceivable that work-related stress could drive some individuals to seek online outlets, highlighting the need for workplace interventions, such as digital wellness programs and structured screen time policies, to mitigate the risk of excessive Internet use (Dipasha Bansal, 2024).

Income status played a pivotal role, as participants whose income was equal to or exceeded their expenses showed a higher propensity for Internet addiction (Balasubramanian & Parayitam, 2023). This might suggest that greater financial security could afford more leisure time or access to digital devices and online platforms, thereby facilitating extended Internet usage. Research indicates that individuals from higher-income backgrounds tend to engage more frequently in online entertainment activities, such as social media, streaming services, and online gaming, which may foster compulsive Internet behaviors (Sahni, 2023). Furthermore, financial stability may reduce the need for strict time management, allowing for increased recreational screen time, which is a known risk factor for Internet addiction (Bakalis et al., 2023). Alternatively, those with lower incomes may experience different stressors and constraints on leisure activities, potentially leading to different patterns of Internet use (Ma et al., 2023). For instance, individuals from lower socioeconomic backgrounds may rely on the Internet for educational or job-seeking purposes rather than for leisure, potentially reducing their risk of addiction (Shalini et al., 2022). However, economic hardships can also contribute to psychological distress, prompting some individuals to seek online escapism as a coping mechanism (Ahmed, 2023). Future research could delve into whether higher-income individuals tend toward specific online domains, such as streaming services or social media platforms, that foster addictive patterns, while lower-income individuals may engage in Internet use differently based on necessity rather than entertainment (Wu & Yao, 2022).

Of note, another important issue to be discussed is that this study focuses on the impact of Internet addiction on psychological well-being and sleep quality. Therefore, it is also crucial to consider the potential for reverse causation. Individuals with pre-existing poor mental health or sleep disturbances may turn to excessive Internet or social media use as a coping mechanism rather than such use being the primary cause of their distress. This perspective is supported by recent research indicating that factors like depressive symptoms can precede and drive social media use rather than the other way around (Hartanto et al., 2021). Consequently, future work should incorporate longitudinal or experimental designs to disentangle these complex bidirectional influences and provide clearer insights into whether Internet addiction leads to—or merely coincides with—deteriorations in mental health and sleep.

One of the key strengths of this study is its focus on adult individuals, a population that has been relatively underexplored in the literature on Internet addiction. By examining the associations between Internet addiction, sleep quality, and psychological well-being in an adult sample, this study contributes valuable insights into how these relationships manifest beyond the commonly studied adolescent and young adult populations. While the current study provides valuable insights, several limitations must be acknowledged. The cross-sectional design precludes establishing causal relationships among Internet addiction, sleep quality, and psychological well-being, as it captures data at a single time point rather than tracking changes over time. Without longitudinal or experimental data, it remains unclear whether Internet addiction leads to poorer sleep and mental health outcomes or whether pre-existing sleep disturbances and psychological distress contribute to problematic Internet use. Additionally, reliance on self-report questionnaires introduces the possibility of social desirability bias, where participants may under-report problematic behaviors or exaggerate positive aspects of their well-being. Furthermore, recall bias may affect the accuracy of responses, particularly regarding sleep patterns and Internet usage habits. Objective measures, such as actigraphy for sleep assessment or digital monitoring tools for Internet use, could provide more reliable data in future research. Another limitation is the sample composition, which consisted solely of participants over the age of 30. This restricts the generalizability of the findings to younger populations, such as adolescents and emerging adults, who often exhibit different Internet use patterns, motivations, and vulnerabilities to addiction. Since younger individuals are more likely to engage in excessive social media use, gaming, or streaming, their risk factors for Internet addiction may differ significantly. Future studies should incorporate more diverse age groups to better understand age-related differences in the impact of Internet addiction on sleep and mental health. Moreover, this study did not account for potential confounding variables such as socioeconomic status, occupational demands, or pre-existing mental health conditions, all of which could influence the observed associations. A more comprehensive approach that includes these factors could help disentangle the complex interplay between Internet use, sleep, and psychological well-being. Future research should employ longitudinal or experimental designs to establish causal relationships and uncover the underlying mechanisms driving these associations. Incorporating neurobiological measures, ecological momentary assessments, and behavioral interventions could further enhance our understanding of Internet addiction and its broader implications for mental health and sleep quality.

## 5. Conclusions

In conclusion, the findings highlight the interconnections among Internet addiction, sleep quality, and psychological well-being. Younger age, female gender, certain employment contexts, and higher income appear to be associated with a heightened risk of problematic Internet use. Although the cross-sectional nature of this study cannot establish causality, the correlations observed between poor sleep quality, diminished psychological well-being, and Internet addiction underline the importance of further research—particularly using longitudinal or experimental designs—to clarify the directionality of these relationships. Tailored interventions—encompassing sleep hygiene education, mental health support, and responsible technology use guidelines—may help mitigate the potential harms associated with excessive Internet use, ultimately fostering healthier behavioral and psychosocial outcomes in adult populations.

## Figures and Tables

**Table 1:** Baseline descriptive and socio-demographic characteristics of the research group.

Variables	Total (n = 629)
Age, mean ± SD, years	39.4 ± 7.8 (min–max: 30–60)
Gender, n (%)	
Female	336 (53.4)
Male	293 (46.6)
Relationship status, n (%)	
Single	188 (29.9)
Not single	441 (70.1)
Educational status, n (%)	
High school and below	208 (33.1)
Undergraduate	309 (49.1)
Postgraduate	112 (17.8)
Working status	
No ^a^	139 (22.1)
Yes ^b^	490 (77.9)
Income status, n (%)	
My income is less than my expenses	252 (40.1)
My income is equal to my expenses	211 (33.5)
My income is more than my expenses	166 (26.4)
Smoking, n (%)	
Yes	151 (24.2)
No	472 (75.8)
Alcohol, n (%)	
Yes ^c^	251 (40.3)
No	372 (59.7)

^a^ I am not working. ^b^ I am working in a job that brings regular income. ^c^ Those who consume alcohol once or more than once a month. SD: standard deviation.

**Table 2:** Total and subscale scores of Internet addiction, psychological well-being, and Pittsburgh sleep quality index (PSQI) scores of the participants (n = 629).

Scale and Subscales	Scores	Minimum Score	Maximum Score
Internet addiction, mean ± SD	38.1 ± 13.6	19	95
Psychological well-being, mean ± SD	43.2 ± 8.8	8	56
PSQI total, mean ± SD	6.9 ± 4.0	0	19
PSQI—subjective sleep quality, mean ± SD	1.3 ± 0.8	0	3
PSQI—sleep onset latency, mean ± SD	1.2 ± 0.9	0	3
PSQI—sleep duration, mean ± SD	0.7 ± 1.0	0	3
PSQI—habitual sleep efficiency, mean ± SD	0.9 ± 1.1	0	3
PSQI—sleep disorders, mean ± SD	1.4 ± 0.6	0	3
PSQI—use of sleep medication, mean ± SD	0.2 ± 0.8	0	3
PSQI—daytime dysfunction, mean ± SD	1.0 ± 0.9	0	3
PSQI, n (%)			
Good sleep (<4)	206 (32.8)		
Poor sleep (≥5)	423 (67.2)		

Internet addiction scale consists of 19 questions and is a five-point Likert type. Psychological well-being consists of 8 questions and is a seven-point Likert type. PSQI total score varies between 0 and 21.

**Table 3:** Relationship between participants’ Internet addiction, psychological well-being, and Pittsburgh sleep quality index (PSQI) total and subscale scores (n = 629).

		Internet Addiction	Psychological Well-Being	PSQI Total	PSQI Subjective Sleep Quality	PSQI Sleep Onset Latency	PSQI Sleep Duration	PSQI Habitual Sleep Efficiency	PSQI Sleep Disorders	PSQI Use of Sleep Medication	PSQI Daytime Dysfunction
Internet addiction	r	1	−0.417 **	0.593 **	0.411 **	0.434 **	0.377 **	0.141 **	0.411 **	0.429 **	0.522 **
	*p*-value		<0.001	<0.001	<0.001	<0.001	<0.001	<0.001	<0.001	<0.001	<0.001
Psychological well-being	r	−0.417 **	1	−0.490 **	−0.400 **	−0.204 **	−0.336 **	−0.051	−0.250 **	−0.598 **	−0.404 **
	*p*-value	<0.001		<0.001	<0.001	<0.001	<0.001	0.203	<0.001	<0.001	<0.001
PSQI total	r	0.593 **	−0.490 **	1	0.721 **	0.631 **	0.743 **	0.539 **	0.627 **	0.564 **	0.693 **
	*p*-value	<0.001	<0.001		<0.001	<0.001	<0.001	<0.001	<0.001	<0.001	<0.001
PSQI—subjective sleep quality	r	0.411 **	−0.400 **	0.721 **	1	0.454 **	0.433 **	0.182 **	0.514 **	0.221 **	0.586 **
	*p*-value	<0.001	<0.001	<0.001		<0.001	<0.001	<0.001	<0.001	<0.001	<0.001
PSQI—sleep onset latency	r	0.434 **	−0.204 **	0.631 **	0.454 **	1	0.323 **	0.179 **	0.386 **	0.216 **	0.332 **
	*p*-value	<0.001	<0.001	<0.001	<0.001		<0.001	<0.001	<0.001	<0.001	<0.001
PSQI—sleep duration	r	0.377 **	−0.336 **	0.743 **	0.433 **	0.323 **	1	0.378 **	0.353 **	0.442 **	0.396 **
	*p*-value	<0.001	<0.001	<0.001	<0.001	<0.001		<0.001	<0.001	<0.001	<0.001
PSQI—habitual sleep efficiency	r	0.141 **	−0.051	0.539 **	0.182 **	0.179 **	0.378 **	1	0.138 **	0.130 **	0.183 **
	*p*-value	<0.001	0.203	<0.001	<0.001	<0.001	<0.001		<0.001	0.001	<0.001
PSQI—sleep disorders	r	0.411 **	−0.250 **	0.627 **	0.514 **	0.386 **	0.353 **	0.138 **	1	0.313 **	0.404 **
	*p*-value	<0.001	<0.001	<0.001	<0.001	<0.001	<0.001	<0.001		<0.001	<0.001
PSQI—use of sleep medication	r	0.429 **	−0.598 **	0.564 **	0.221 **	0.216 **	0.442 **	0.130 **	0.313 **	1	0.298 **
	*p*-value	<0.001	<0.001	<0.001	<0.001	<0.001	<0.001	0.001	<0.001		<0.001
PSQI—daytime dysfunction	r	0.522 **	−0.404 **	0.693 **	0.586 **	0.332 **	0.396 **	0.183 **	0.404 **	0.298 **	1
	*p*-value	<0.001	<0.001	<0.001	<0.001	<0.001	<0.001	<0.001	<0.001	<0.001	

** Correlation is significant at the 0.01 level (2-tailed).

**Table 4:** Multiple linear regression analysis scrutinizing the effects of comparable variables on changes in Internet addiction scale scores.

Predictor *	Unstandardized Coefficients Beta	Coefficients Std. Error	Standardized Coefficients Beta	t	95% CI for Beta	*p*-Value **
R = 0.721 **; R square = 0.520 **						
Age	−0.322	0.055	−0.184	−5.822	−0.431–0.214	**<** **0.001 ^†^**
Gender	−2.403	0.821	−0.088	−2.928	−4.015–−0.791	**0.004 ^†^**
Working status	2.686	1.145	0.082	2.346	−0.438–4.934	**0.019 ^†^**
Educational status (Undergraduate)	−1.969	0.871	−0.072	−2.261	−3.679–−0.259	**0.024 ^†^**
Income status (My income is equal to my expenses)	5.078	0.940	0.176	5.401	3.232–6.925	**<0.001 ^†^**
Income status (My income is more than my expenses)	4.250	1.030	0.136	4.125	2.227–6.274	**<0.001 ^†^**
PSQI total	3.598	0.322	1.067	11.178	2.966–4.230	**<0.001 ^†^**
PSQI—subjective sleep quality	−3.456	0.773	−0.216	−4.469	−4.975–−1.937	**<0.001 ^†^**
PSQI—sleep onset latency	−1.192	0.644	−0.080	−1.851	−2.457–0.073	0.065
PSQI—sleep duration	−1.798	0.660	−0.133	−2.725	−3.093–−0.502	**0.007 ^†^**
PSQI—habitual sleep efficiency	−4.126	0.486	−0.352	−8.482	−5.081–−3.171	**<0.001 ^†^**

* Multiple linear regression included variables with a *p* < 0.100 criterion and was calculated by using the backward linear regression method. ** Seven steps. CI: confidence interval. Reference for gender is “female”; Reference for Working status is “None”; Reference for smoking and alcohol is “None”; ^†^ Statistically significant.

## Data Availability

The datasets used and/or analyzed in this study are available upon reasonable request from the corresponding author.
